# Radiomics feature robustness as measured using an MRI phantom

**DOI:** 10.1038/s41598-021-83593-3

**Published:** 2021-02-17

**Authors:** Joonsang Lee, Angela Steinmann, Yao Ding, Hannah Lee, Constance Owens, Jihong Wang, Jinzhong Yang, David Followill, Rachel Ger, Dennis MacKin, Laurence E. Court

**Affiliations:** 1 Department of Radiation Physics, Unit 1420, The University of Texas MD Anderson Cancer Center, 1515 Holcombe Boulevard, Houston, TX 77030 USA; 2 Department of Computational Medicine and Bioinformatics, University of Michigan, 500 S State Street, Ann Arbor, MI 48109 USA

**Keywords:** Image processing, Outcomes research

## Abstract

Radiomics involves high-throughput extraction of large numbers of quantitative features from medical images and analysis of these features to predict patients’ outcome and support clinical decision-making. However, radiomics features are sensitive to several factors, including scanning protocols. The purpose of this study was to investigate the robustness of magnetic resonance imaging (MRI) radiomics features with various MRI scanning protocol parameters and scanners using an MRI radiomics phantom. The variability of the radiomics features with different scanning parameters and repeatability measured using a test–retest scheme were evaluated using the coefficient of variation and intraclass correlation coefficient (ICC) for both T1- and T2-weighted images. For variability measures, the features were categorized into three groups: large, intermediate, and small variation. For repeatability measures, the average T1- and T2-weighted image ICCs for the phantom (0.963 and 0.959, respectively) were higher than those for a healthy volunteer (0.856 and 0.849, respectively). Our results demonstrated that various radiomics features are dependent on different scanning parameters and scanners. The radiomics features with a low coefficient of variation and high ICC for both the phantom and volunteer can be considered good candidates for MRI radiomics studies. The results of this study will assist current and future MRI radiomics studies.

## Introduction

Medical imaging plays an important role in clinical cancer care for diagnosis, radiation therapy, treatment planning, and cancer management. Researchers have developed various analytical medical imaging methods, such as image segmentation, registration, pattern recognition, and multivariate pattern classification. One of these, radiomics^1–4^, has recently emerged as a promising medical image analysis tool for diagnosis and prediction of response to treatment of various diseases. Radiomics involves the high-throughput extraction of large numbers of quantitative features from medical images and analysis of these features to predict patients’ outcome and support clinical decision-making, such as classifying benign and malignant tumors, determining molecular subtypes and/or mutation status, and predicting overall survival.

Several radiomics analyses have been used with various imaging modalities in oncology, such as computed tomography (CT), magnetic resonance imaging (MRI), and positron emission tomography (PET), and results showed that a large number of radiomics features have prognostic power in several studies, such as lung and head and neck cancer patients on CT images^3,5^, prognosis of recurrence and survival in lung cancer patients on PET/CT images^6,7^, and in brain tumor and breast cancer patients on MRI images^8–12^. Radiomics features are sensitive to several factors, however, such as reconstruction settings^13,14^, tumor delineation^15^, scanning protocols^16,17^, different scanners^18^, and various noise sources. Several radiomics studies have investigated reproducibility and repeatability^19^. For example, Peerlings et al.^20^ investigated on stability of radiomics features in apparent diffusion coefficient (ADC) maps. Schwier et al.^21^ investigated on repeatability of multiparametric prostate MRI radiomics features. Fave et al.^22^ evaluated how different image preprocessing techniques may impact both the volume dependence and prognostic potential of the features of non-small cell lung cancer in CT and investigated the variability in voxel size, slice thickness, and convolution kernels in CT^23^. Also, Mackin et al.^24^ investigated variability in radiomics features with the x-ray tube current used in CT. In a recent study, Shiri et al.^25^ investigated the impact of image reconstruction settings on radiomics features using two PET/CT scanners. They found that the variability and robustness of PET/CT images are dependent on different features and concluded that radiomics features with a low coefficient of variation (COV) are good candidates for reproducible tumor quantification in multicenter studies. In a similar study of PET, Bailly et al.^26^ investigated the variability of 15 textural features according to reconstruction parameters in multicenter trials and found that Homogeneity, Entropy, Dissimilarity, High Gray-Level Run Emphasis (HGRE), High Gray Level-Zone Emphasis (HGZE), and Zone Percentage (ZP) features are robust and suitable for use in multicenter trials.

However, not many studies have investigated the repeatability (variations when a patient is scanned twice on the same scanner with the same parameters) and variability when different scanning protocols are used for MRI radiomics studies. MRI is an important diagnostic imaging modality and has been widely used as a major diagnostic tool in both clinical imaging and scientific research, and quantitative radiomics analysis using MRI has increased recently.

Therefore, in the present study, we created an MRI radiomics phantom and used it to assess the robustness of MRI radiomics features with various MRI scanning protocols and two MRI scanners. First, we evaluated radiomics features of the MRI phantom by comparing each feature value with patient population data using the two-sigma range of feature values extracted from 97 T1- and T2-weighted MR images of patients with brain lesions. We then investigated the robustness of magnetic resonance imaging (MRI) radiomics features with various MRI scanning protocol parameters and scanners using an MRI radiomics phantom.

## Results

**Figure 1:**
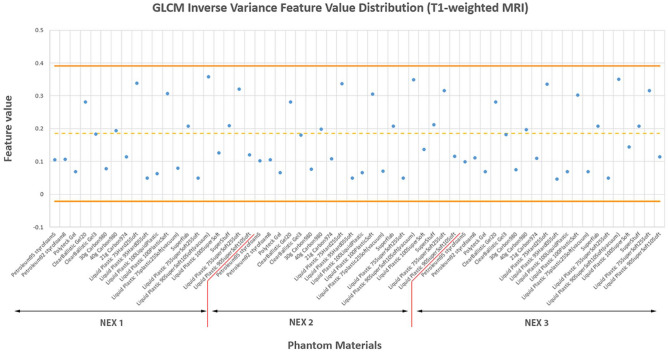
GLCM inverse variance feature values for the MRI phantom materials with various settings in NEX. The orange solid lines and orange dashed line are the mean ± 2 SDs bounds and the mean of the patient population data for the inverse variance feature, respectively.

We determined the suitability of the MRI phantom materials by comparing the radiomics feature values from the phantom materials with those of the brain lesions of the patient data (mean values ± two standard deviations [SDs]) (Table 1). Figure 1 illustrates this analysis, showing the values of the inverse variance texture feature for the phantom materials over various settings in a number of excitations (NEX). The orange solid lines and orange dashed line in the figure represent the mean ± two SDs bounds and mean patient population data for the inverse variance feature, respectively. Averages of 92.5% and 79.6% phantom radiomics features for the 20 materials were within the established patient population bounds for T1- and T2-weighted images, respectively.

**Table 1:** The percentages of radiomics features for the MRI phantom within the established patient population bounds (mean ± 2 SDs).

Scanner		NEX	ST	FOV	Matrix	Average (%)
Siemens 1	T1	90.7	91.2	89.3	90.9	90.5
	T2	79.8	80.3	83.3	NA	81.1
Siemens 2	T1	90.9	91.1	91.3	89.6	90.7
	T2	90.9	80.4	84.0	NA	85.1
Phillips	T1	96.0	97.2	95.8	NA	96.3
	T2	80.0	67.9	69.3	NA	72.4
Average	T1					92.5
	T2					79.6

Siemens 1 and 2 represent two repeated scans in a Siemens scanner; *ST* slice thickness, *FOV* field of view, *NA* not available.

**Figure 2:**
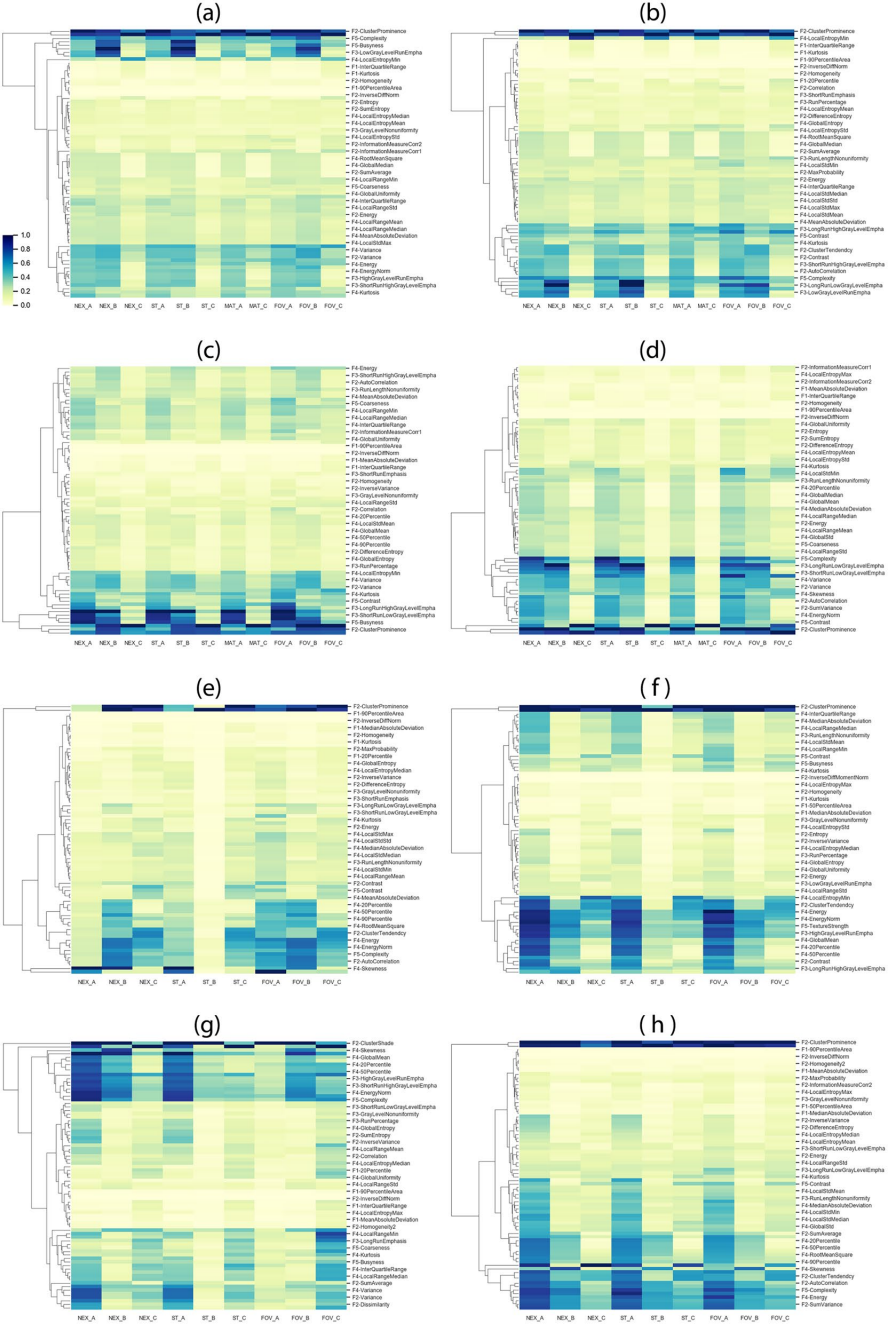
Heat map of the variability of radiomics features with different MRI scanning settings for (**a**) T1-weighted and (**b**) T2-weighted images without normalization and filtering. (**c**,**d**) shows the heat map for T1- and T2-weighted images with normalization. (**e**,**f**) shows the heat map for T1- and T2-weighted images with filtering, respectively. We also test the variability of radiomics features with different ROI size (regular ROI = diameter of 1.8 cm and small ROI = diameter of 1.2 cm). (**g**,**h**) shows the heat map for T1- and T2-weighted images with smaller ROI size. On the x-axis, _A, _B, and _C represent phantom scanned using the Siemens 1.5 T scanner, phantom scanned using the Philips 1.5 T scanner, and volunteer scanned using the Siemens 1.5 T scanner, respectively. The COV value is rescaled from 0 to 1 with blue representing high COV values. The hierarchical clustering on the y-axis was computed using a Euclidean distance measure. The full feature names for each plot listed in the tables S13, S14, S15, and S16 in the supplementary information.

We used the COV to assess the variability of radiomics features for the impact of different MRI parameter settings and plotted a heat map of the COV for both the phantom and volunteer. We repeated this with image intensity normalization, without normalization, with smoothing filter, and without smoothing filter as a preprocessing, respectively (Fig. 2). We used 3 sigma method^27^ for the intensity normalization and the butterworth algorithm^28–30^ for the smoothing filter. We also investigated the variability of radiomics features with different ROI size (diameter of 1.2 cm) (Fig. 2). Based on the COV, we categorized the features in terms of variation using three groups: large variation (COV > 30%), intermediate variation (10% < COV ≤ 30%), and small variation (COV ≤ 10%)^25^. Without any image reconstruction such as normalization and filtering process, the average COVs in these three groups were 6.1%, 18.5%, and 45.5%, respectively, for T1-weighted images and 4.5%, 17.2%, and 51.4%, respectively, for T2-weighted images. Tables 2 and 3 summarize the radiomics features in the three groups for T1- and T2-weighted images, respectively. With normalization and filtering process, the average COVs for three groups summarized in Table 4. The detailed radiomics features in the three groups for T1 and T2- weighted images are listed in Tables S4, S5, S6, S7, S8 and S9 in the supplement information.

**Table 2:** Variations of radiomics features over different MRI scanning settings for T1-weighted images without normalization and smoothing.

Feature category	COV < 10%	10% ≤ COV < 30%	COV ≥ 30%
Gradient orient histogram(7)	InterQuartileRange	90PercentileArea	
	Kurtosis		
	MeanAbsoluteDeviation		
	MedianAbsoluteDeviation		
	20Percentile		
	50PercentileArea		
Gray level cooccurence (22)	InverseDiffMomentNorm	AutoCorrelation	ClusterProminence
	DifferenceEntropy	Contrast, Correlation	ClusterShade
	Homogeneity	Dissimilarity	ClusterTendendcy
	InformationMeasureCorr2	Energy, Entropy	SumVariance
	Homogeneity2	InformationMeasureCorr1	Variance
	InverseDiffNorm	MaxProbability	
	InverseVariance	SumAverage	
	SumEntropy		
Gray level run length (11)	GrayLevelNonuniformity	HighGrayLevelRunEmpha	LongRunHighGrayLevelEmpha
	ShortRunEmphasis	LongRunEmphasis	LongRunLowGrayLevelEmpha
		RunLengthNonuniformity	LowGrayLevelRunEmpha
		RunPercentage	ShortRunHighGrayLevelEmpha
			ShortRunLowGrayLevelEmpha
Intensity (31)	GlobalEntropy	GlobalMean	Energy
	LocalEntropyMean	GlobalMedian, GlobalStd	EnergyNorm
	LocalEntropyMax	GlobalUniformity	LocalEntropyMin
		InterQuartileRange	Skewness
		Kurtosis, LocalEntropyStd	Variance
		LocalEntropyMedian	
		LocalRangeMean	
		LocalRangeMin	
		LocalRangeStd	
		LocalStdMax, LocalStdMean	
		LocalStdMedian, LocalStdMin	
		LocalStdStd	
		MeanAbsoluteDeviation	
		MedianAbsoluteDeviation	
		20Percentile, 50Percentile	
		90Percentile	
		RootMeanSquare	
		LocalRangeMedian	
Neighborhood gray-tone difference (NGTD) (5)		Coarseness	Busyness
			Complexity
			Contrast
			TextureStrength
Average COV	6.05%	18.52%	45.49%

**Table 3:** Variations of radiomics features over different MRI scanning settings for T2-weighted images without normalization and smoothing.

Feature category	COV < 10%	10% ≤ COV < 30%	COV ≥ 30%
Gradient orient histogram	InterQuartileRange		
	Kurtosis		
	MeanAbsoluteDeviation		
	MedianAbsoluteDeviation		
	20Percentile		
	50PercentileArea		
	90PercentileArea		
Gray level cooccurence	DifferenceEntropy	Correlation	AutoCorrelation
	Homogeneity	Dissimilarity	ClusterProminence
	Homogeneity2	Energy	ClusterShade
	InformationMeasureCorr2	Entropy	ClusterTendendcy
	SumEntropy	InformationMeasureCorr1	Contrast
	InverseVariance	MaxProbability	SumVariance
	InverseDiffMomentNorm	SumAverage	Variance
	InverseDiffNorm		
Gray level run length	GrayLevelNonuniformity	LongRunEmphasis	HighGrayLevelRunEmpha
	RunPercentage	LongRunLowGrayLevelEmpha	LongRunHighGrayLevelEmpha
	ShortRunEmphasis	LowGrayLevelRunEmpha	ShortRunHighGrayLevelEmpha
		RunLengthNonuniformity	
		ShortRunLowGrayLevelEmpha	
Intensity	GlobalUniformity	GlobalEntropy	Energy
	LocalEntropyMax	GlobalStd	EnergyNorm
	LocalEntropyMean	InterQuartileRange	GlobalMean
	LocalEntropyStd	LocalEntropyMedian	GlobalMedian
	LocalStdMax	LocalRangeMean	LocalEntropyMin
		LocalRangeMedian	20Percentile
		LocalRangeMin	50Percentile
		LocalRangeStd	90Percentile
		LocalStdMean	RootMeanSquare
		LocalStdMedian	Variance
		LocalStdMin	
		LocalStdStd	
		MeanAbsoluteDeviation	
		MedianAbsoluteDeviation	
		Kurtosis	
		Skewness	
Neighborhood gray-tone difference (NGTD) (5)		Busyness	Complexity
		Coarseness	TextureStrength
		Contrast	
Average COV	4.49%	17.15%	51.41%

**Table 4:** Average COV for T1- and T2-weighted images.

	COV < 10%	10% ≤ COV < 30%	COV ≥ 30%
**T1-weighted images**			
No preprocessing	6.05%	18.52%	45.49%
No preprocessing (small ROI)	4.59%	17.80%	45.73%
Normalization	4.87%	16.83%	48.32%
Filtering	4.29%	17.72%	45.09%
**T2-weighted images**			
No preprocessing	4.49%	17.15%	51.41%
No preprocessing (small ROI)	5.67%	17.41%	45.68%
Normalization	4.67%	19.35%	45.92%
Filtering	4.69%	19.66%	52.16%

*Small ROI* diameter of 1.2 cm, *regular ROI* diameter of 1.8 cm.

**Figure 3:**
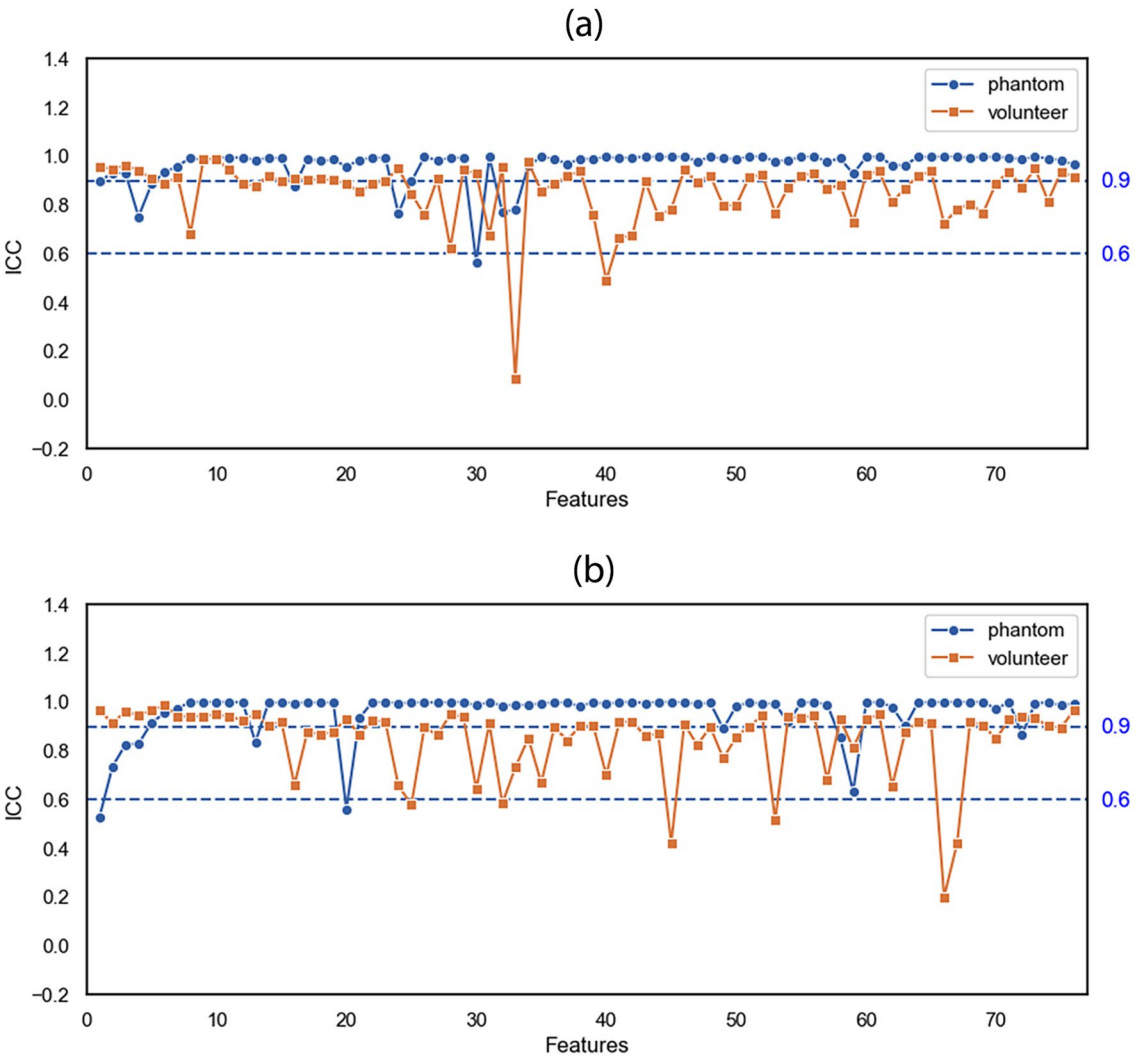
ICC plots in (**a**) T1-weighted and (**b**) T2-weighted images of the phantom and volunteer. The blue circles and orange squares represent the ICC values for the phantom and volunteer, respectively. The order of the features’ names is shown in Table 9.

Figure 3 shows intraclass correlation coefficient (ICC) plots for T1- and T2-weighted images of the phantom and volunteer for a test–retest scheme on a single scanner. We found that the T1- and T2-weighted image repeatability measures for the phantom (average ICC, 0.963 and 0.959, respectively) were higher than those for the volunteer (average ICC, 0.856 and 0.849, respectively). In this study, we categorized repeatability variations using three groups: high repeatability (ICC ≥ 0.9), intermediate repeatability (0.6 ≤ ICC < 0.9), and poor repeatability (ICC < 0.6)^31^. Tables 5 and 6 summarize the repeatability of the radiomics features for various MRI scanning parameters for all three groups for the phantom and volunteer, respectively. For the phantom, the ICC for all features except the Gray Level Non-uniformity (T1), Inter Quartile Range (T2), and Information Measure Corr 1 (T2) was greater than 0.6 for both T1- and T2-weighted images. For the feature comparison between with and without normalization, with and without smoothing effects, and different ROI sizes, we summarized the results in Tables S10, S11, S12, respectively. Based on these results, we can see that features in GLRL and NID are more invariant compared to other feature categories.

**Table 5:** Repeatability of the radiomics features with different MRI scanning settings using the same scanner for the phantom.

	ICC < 0.6	**0.6 ≤ ICC < 0.9**	**ICC ≥ 0.9**
T1	**GrayLevelRunLengthMatrix25(GLRLM):** GrayLevelNonuniformity	**GradientOrientHistogram:** MedianAbsoluteDeviation InterQuartileRange 20Percentile **Texture(GLCM):** Energy, InverseVariance MaxProbability **GLRLM:** LongRunEmphasis LongRunHighGrayLevelEmpha	**GradientOrientHistogram:** Kurtosis, MeanAbsoluteDeviation 50PercentileArea, 90PercentileArea **Texture(GLCM):** AutoCorrelation, ClusterProminence ClusterShade, ClusterTendendcy Contrast, DifferenceEntropy, Correlation Dissimilarity, Entropy, Homogeneity Homogeneity2, InformationMeasureCorr1 InformationMeasureCorr2, SumAverage InverseDiffMomentNorm, SumEntropy SumVariance, Variance **GrayLevelRunLengthMatrix25(GLRLM):** HighGrayLevelRunEmpha, ShortRunEmphasis LongRunLowGrayLevelEmpha, RunLengthNonuniformity LowGrayLevelRunEmpha, RunPercentage ShortRunHighGrayLevelEmpha, ShortRunLowGrayLevelEmpha **Intensity:** Energy, EnergyNorm, GlobalEntropy, GlobalMean GlobalMedian, GlobalStd, GlobalUniformity, Kurtosis, InterQuartileRange, LocalEntropyMax, LocalRangeMedian, LocalEntropyMean, LocalEntropyMedian, LocalRangeMin LocalEntropyMin, LocalEntropyStd, Variance LocalRangeMean, LocalRangeStd, LocalStdMax, LocalStdMean, Skewness, LocalStdMedian, LocalStdMin, LocalStdStd, MeanAbsoluteDeviation, RootMeanSquare MedianAbsoluteDeviation 20Percentile, 50Percentile, 90Percentile **Neighborhood Gray-Tone Difference (NGTD):** Busyness, Coarseness, Complexity Contrast, TextureStrength
T2	**GradientOrientHistogram:** InterQuartileRange **Texture(GLCM):** InformationMeasureCorr1	**GradientOrientHistogram:** Kurtosis MeanAbsoluteDeviation MedianAbsoluteDeviation **Texture(GLCM):** Correlation **Intensity:** Kurtosis, LocalStdMax LocalRangeStd **NeighborIntensityDifference:** Busyness	**GradientOrientHistogram:** 20Percentile, 50PercentileArea 90PercentileArea **Texture(GLCM):** AutoCorrelation, ClusterShade ClusterProminence, Contrast ClusterTendendcy, Energy, Variance DifferenceEntropy, Entropy Dissimilarity, Homogeneity Homogeneity2, InverseDiffNorm InformationMeasureCorr2 InverseDiffMomentNorm, SumEntropy InverseVariance, MaxProbability SumAverage, SumVariance **Texture (GLRLM):** GrayLevelNonuniformity, RunPercentage HighGrayLevelRunEmpha, ShortRunEmphasis LongRunEmphasis, ShortRunHighGrayLevelEmpha LongRunHighGrayLevelEmpha LongRunLowGrayLevelEmpha LowGrayLevelRunEmpha RunLengthNonuniformity ShortRunLowGrayLevelEmpha **Intensity:** Energy, EnergyNorm, GlobalEntropy GlobalMean, GlobalMedian, GlobalStd GlobalUniformity, InterQuartileRange LocalEntropyMax, LocalEntropyMean LocalEntropyMedian, LocalEntropyMin LocalEntropyStd, LocalRangeMean LocalRangeMedian, LocalRangeMin LocalStdMean, LocalStdMedian LocalStdStd, MeanAbsoluteDeviation MedianAbsoluteDeviation, 20Percentile 50Percentile, 90Percentile, RootMeanSquare Skewness, Variance, LocalStdMin **Neighborhood Gray-Tone Difference (NGTD):** Coarseness, Complexity, Contrast TextureStrength

**Table 6:** Repeatability of the radiomics features with different MRI scanning settings using the same scanner for the volunteer.

	ICC < 0.6	0.6 ≤ ICC < 0.9	ICC ≥ 0.9
T1	**GrayLevelRunLengthMatrix(GLRLM):** LongRunHighGrayLevelEmpha ShortRunLowGrayLevelEmpha	**GradientOrientHistogram:** 50PercentileArea **Texture (GLCM):** AutoCorrelation, Contrast, Correlation Dissimilarity, InverseDiffNorm InformationMeasureCorr1, MaxProbability InformationMeasureCorr2, SumAverage InverseDiffMomentNorm, SumVariance **Texture (GLRLM):** HighGrayLevelRunEmpha ShortRunHighGrayLevelEmpha **Intensity:** Energy, EnergyNorm, GlobalEntropy GlobalMean, GlobalMedian, Kurtosis LocalEntropyMax, LocalEntropyMin LocalEntropyStd, LocalRangeMin LocalRangeStd, LocalStdMax, LocalStdMin LocalStdStd, 20Percentile, 50Percentile 90Percentile, RootMeanSquare, Skewness **Neighborhood Gray-Tone Difference (NGTD):** Busyness, Complexity	**GradientOrientHistogram:** InterQuartileRange, 20Percentile Kurtosis, 90PercentileArea MeanAbsoluteDeviation MedianAbsoluteDeviation **Texture (GLCM):** ClusterProminence, ClusterShade ClusterTendendcy, DifferenceEntropy Energy, Entropy, Homogeneity Homogeneity2, InverseVariance SumEntropy, SumEntropy, Variance **GrayLevelRunLengthMatrix (GLRLM):** GrayLevelNonuniformity LongRunEmphasis, LongRunLowGrayLevelEmpha RunPercentage, ShortRunEmphasis **Intensity:** GlobalStd, InterQuartileRange LocalEntropyMean, LocalEntropyMedian LocalRangeMean, LocalRangeMedian LocalStdMean, LocalStdMedian MeanAbsoluteDeviation, Variance MedianAbsoluteDeviation **Neighborhood Gray-Tone Difference (NGTD):** Coarseness, Contrast, TextureStrength
T2	**Texture(GLCM):** MaxProbability **Texture (GLRLM):** LongRunEmphasis **Intensity:** GlobalMedian LocalEntropyMin 20Percentile 50Percentile **IntensityHistogram:** 20Percentile 50Percentile	**Texture(GLCM):** Energy, Entropy InverseVariance Homogeneity Homogeneity2 InformationMeasureCorr2 SumAverage, SumEntropy **Texture (GLRLM):** GrayLevelNonuniformity LongRunHighGrayLevelEmpha LongRunLowGrayLevelEmpha LowGrayLevelRunEmpha RunLengthNonuniformity RunPercentage ShortRunLowGrayLevelEmpha **Intensity:** GlobalEntropy, GlobalMean GlobalUniformity, Kurtosis InterQuartileRange LocalEntropyMax, variance LocalStdMax, LocalStdMin LocalStdStd, Skewness **Neighborhood Gray-Tone Difference (NGTD):** Contrast	**GradientOrientHistogram:** InterQuartileRange Kurtosis, 20Percentile MeanAbsoluteDeviation MedianAbsoluteDeviation 50PercentileArea, 90PercentileArea **Texture (GLRLM):** AutoCorrelation, SumVariance ClusterProminence, Contrast ClusterShade, Dissimilarity ClusterTendendcy, Variance DifferenceEntropy, Correlation InformationMeasureCorr1 InverseDiffMomentNorm InverseDiffNorm **Texture (GLRLM):** HighGrayLevelRunEmpha ShortRunEmphasis ShortRunHighGrayLevelEmpha **Intensity:** Energy, EnergyNorm, GlobalStd LocalEntropyMean, LocalRangeStd LocalEntropyMedian, Variance LocalEntropyStd, LocalStdMean LocalRangeMean, LocalStdMedian LocalRangeMedian, 90Percentile MeanAbsoluteDeviation MedianAbsoluteDeviation RootMeanSquare **Neighborhood Gray-Tone Difference (NGTD):** Busyness, Coarseness Complexity, TextureStrength

## Discussion

**Figure 4:**
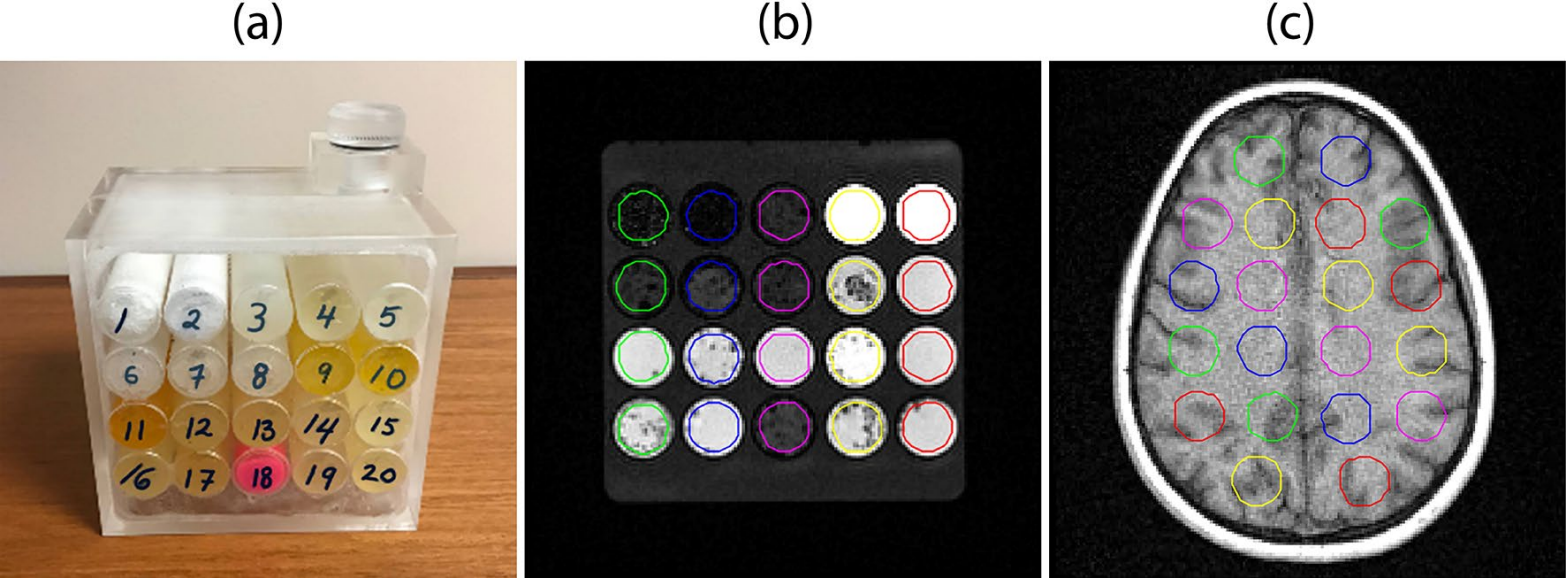
The MRI phantom and the healthy volunteer’s brain (**a**) The MRI phantom consisting of 20 different materials. (**b**) A T1-weighted image of the phantom. (**c**) A T1-weighted image of the healthy volunteer’s brain with 20 ROIs.

In recent years, radiomic studies have become increasingly important for medical image analysis to assist the diagnosis, prognosis, and prediction of treatment response within clinical-decision making systems. However, radiomics features are sensitive to different image reconstruction settings, scanning protocols, scanners, and noise sources, so we must identify the radiomics features that remain stable to provide accurate and reliable decision support for patient care. In the present study, we made our phantom with 20 homogeneous and heterogeneous materials selected carefully (Fig. 4B). So, our phantom is similar to the human brain as brain has both homogeneous and heterogeneous regions for fair comparison. We showed the suitability of the phantom materials by comparing radiomics features obtained from phantom materials with those of the brain lesions of patients. We used the brain MRI data over other patient anatomies because of its stable movement. Various studies showed that respiratory motion was a major factor leading to irreproducibility in various modalities such as MRI, PET, and CT^32^. Next, we investigated the variability and repeatability in radiomics features extracted from T1- and T2-weighted MR images of an MRI phantom and a healthy volunteer to identify radiomics feature robustness for various scanning protocols and different scanners. Our results showed that the robustness of the MRI radiomics features across the different scanning protocols varies depending on radiomics features. According to our results, most intensity-based and gray level co-occurrence matrix (GLCM) features were in the intermediate or small variation group, whereas most neighborhood gray-tone difference (NGTD) features were in the high variation group. NGTD features are extracted from an image inside the region of interest (ROI), and intensity difference is computed in a two-dimensional neighborhood. NGTD features provide fundamental texture properties, such as coarseness, contrast, busyness, complexity, and texture strength^33^. Of the GLCM features, variance, cluster shade, cluster tendency, and cluster prominence varied highly across different MRI scanning settings for both the volunteer and phantom, implying that these features are associated with poor robustness. Yang et al.^34^ investigated on the impact of contouring variability on PET radiomics features in the lung. They reported that the impact of contouring variability is present to varying degrees. In this study, we used the same uniform ROI size for both the volunteer and phantom. Our results showed that some features vary more than other features with different settings. The reason is that each feature has its own formula to express its characteristics of the image and some features are dealing with pixel-wise changes such as NGTD features that describe the differences between each voxel and the neighboring voxels, while other features are dealing with overall (average) changes in an image such as sum average that quantify the mean of the sum histogram of an image. Although NGTD features and these four GLCM features are sensitive to different scanning parameters, they have high reproducibility if the parameters are kept the same. These features, therefore, may be useful for intrascanner studies with fixed protocol settings.

In this study, we performed several scans with various scanning protocol parameters such as NEX, slice thickness, phasing steps, and FOV for T1 and T2 respectively with a multi-center scanner. We also performed all scans twice for each setting to evaluate the reliability of scans. Although we limited the number of scans, our results of repeatability showed highly reproducible. For the repeatability measures, we computed the ICC for radiomics features obtained using the two MRI scanners and showed that the repeatability for the phantom was very high (average ICC, 0.963 and 0.959 for T1- and T2-weighted images, respectively) but that the repeatability for the volunteer was intermediate (average ICC, 0.865 and 0.849 for T1- and T2-weighted images, respectively). The repeatability of the volunteer is slightly lower than that of the phantom. This is not surprising, as humans have factors such as patient movement, respiration, and blood flow that can affect radiomics features, and also highlights the fact that phantom measurements alone are not sufficient for understanding variabilities in MRI-based radiomics features. Also, we showed that for the volunteer, the overall repeatability for T1-weighted images was slightly lower than that for T2-weighted images. Of note is that 39 radiomics features were highly reproducible for T1-weighted images of the volunteer, and 41 radiomics features were highly reproducible for T2-weighted images. The variability results for the normalization and filtering effect (Table 4) did not show much difference between them in average COV values.

We also found that radiomics features have different effects depending on the scanning parameters, which similar studies by other groups also demonstrated. For example, Ford et al.^35^ investigated the impact of pulse sequence parameter selection (i.e., echo time [TE] and repetition time [TR]) on MRI textural features of the brain. They found that the variability in radiomics features with the choice of pulse sequence and imaging parameters was feature-dependent and can be substantial. In another study, Saha et al.^17^ assessed the impact of various MRI scanner parameters on the radiomics features in breast MRI studies. They found that the feature group related to variation in fibroglandular tissue enhancement was the most sensitive to the scanner manufacturer and parameters.

Our study had some limitations. First, we could not remove the effect of the volunteer’s movement including blood flow, which influences radiomics feature values. We sought to minimize this effect by using an immobilization mask to fix the volunteer’s head in place during the scan. Also, we simulated a movement effect with the phantom on an MR image. For example, we shifted an image 1 mm to the right and generated a new image by averaging this shifted image with the original image to simulate an image for NEX 2. However, this simulation study did not change the radiomics feature values and does not explain the effect of the volunteer’s motion artifacts including blood flow. For repeatability measures, we took about a 30-min break between two scans for the volunteer. This may have resulted in uncertainties when the volunteer returned to the original position. In this study, we performed image preprocessing to reduce uncertainty in the feature analysis and used a uniform ROI size. However, there is an uncertainty remaining in the lesion segmentation procedure of the patient data, which may affect the suitability test for our phantom materials. Lastly, it should be noted that our previous study and other work reported volume-dependent and gray level-dependent features^22,36^, respectively. In the current study, Tables S2 and S3 are provided in the supplementary information to show the corrected formulas along with the original formulas for the volume-dependent and the gray level-dependent GLCM features, respectively. In this study, corrected formulas were used for the volume-dependent features (Table S2) but original formulas were used for the gray level-dependent GLCM features (Table S3). Please note that since our analysis is based on the same gray levels with various MRI parameter settings for GLCM features, different gray levels with different MRI parameter settings could have different results although the GLCM features in the large variation (COV > 30%) would still be in the same category. Also, it should be noted that our repeatability test will not be affected since the repeatability analysis used the same parameter settings.

In this study, we aimed to identify the robustness of MRI radiomics features with various scanning parameters and multi-scanner variation using an MRI radiomics phantom, which is very useful for calibrating, testing, and evaluating new MRI techniques and variability and repeatability measurements. In this study, we focused on the scanning parameters such as NEX, slice thickness, phasing steps, and FOV, which are the most commonly used in MRI scanning and we fixed all other parameters including filtering, smoothing, and coil sensitivity to avoid introducing other uncertainty factors in this study. We showed that all of the materials in the phantom were suitable by comparing its radiomics features with the patient data from the 97 T1- and T2-weighted MR images and investigated the robustness of various radiomics features with different MRI scanning protocols and two scanners.

## Conclusions

In the present work, an MRI phantom was constructed with 20 MRI materials covering a wide range of radiomics feature values and several scans were performed with various scanning protocol parameters such as NEX, slice thickness, phasing steps, and FOV for T1 and T2 respectively. The ICC showed high repeatability for the phantom but intermediate repeatability for the volunteer, while the COV revealed little difference in variability between normalization and filtering effect.

We believe that this study is very useful for practice in the radiomics community, especially in MRI radiomics studies. Our results demonstrated that various radiomics features have different effects depending on the different scanning parameters and scanners. Furthermore, we identified the robust MRI radiomics features with various scanning parameters and multi-scanner variation using an MRI radiomics phantom. The radiomics features with a low COV and high ICC can be considered good candidates for MRI radiomics studies, whereas those with a high COV and low ICC must be used with caution.

## Methods

### MRI phantom and volunteer

An MRI phantom was created and used to investigate the repeatability and robustness in quantitative radiomics features with various MRI scanning protocol parameters, preprocessing (normalization and image filtering), and scanners. Figure 4 shows the MRI phantom, which was made of acrylic with dimensions of 14.5 × 17.8 × 10.3 cm. Inside the phantom, there were 20 cylinders and each cylinder had a diameter of 2.4 cm and length of 10.3 cm. The phantom could be filled with water through the hole on top of it (Fig. 4A). The MRI phantom was constructed of 20 MRI materials covering a wide range of radiomics feature values (Table 7).

**Table 7:** The 20 materials used in the MRI phantom.

Materials	
1. 5% Styrofoam Ball & 95% Petroleum Jelly 2. 8% Styrofoam Ball & 92% Petroleum Jelly 3. Polyteck Gel 00 4. ClearBallistic Gell 20 5. ClearBallistic Gell 3 6. 30 g Carbon 980 & 300 ml h20 7. 40 g Carbon 980 & 180 ml h20 8. 21 g Carbon 974 & 220 ml h20 9. Liquid Plastic: 75% Hardener & 25% Softener 10. Liquid Plastic: 95% Hardener & 5% Softener	11. Liquid Plastic: 100% liquid plastic 12. Liquid Plastic: 75% plastic soft 13. Liquid Plastic: 75% plastic & 25% Softener 14. Superflab 15. Liquid Plastic: 75% SuperSoft & 25% Softener 16. Liquid Plastic: 90% SuperSoft & 10% Softener 17. Liquid Plastic: 100% SuperSoft plastic 18. SuperStuff 19. Liquid Plastic: 75% SuperSoft & 25% Softener 20. Liquid Plastic: 90% SuperSoft & 10% Softener

The phantom and the brain of the healthy volunteer were scanned using a 1.5 T Siemens MRI system (SIEMENS Magnetom Aera, Erlangen, Germany) with three-dimensional T1-weighted gradient echo sequence and T2-weighted fast spin echo sequence. A fixed TR (11 ms) and TE (4.77 ms) and flip angle of 30° with various scanning protocol parameters were used for T1-weighted images. For T2-weighted images, a TE of 281 ms, TR of 1530 ms, and flip angle of 160° with various scanning protocols were used. For comparison, scanning of the MRI phantom was also performed using a 1.5 T Philips MRI system (PHILIPS Marlin, Finland). For this scanner, a fixed TR (11 ms) and TE (4.61 ms) and flip angle of 30° were used for T1-weighted images, and a TE of 281 ms, TR of 1535 ms, and flip angle of 90° were used for T2-weighted images. We then varied the following scanning protocol parameters: number of excitation (NEX), slice thickness, phasing steps, and field of view (FOV). The detailed scanning protocols are listed in Table 8. Each scan was performed twice with the same setting for both scanners for the repeatability test. The phantom was removed from the scanner after the first scan and repositioned for the second scan. For the volunteer, the scan was also performed twice with the same setting and the volunteer took about a 30-min break between the two scans. The scans were performed each week for multi-scanner variability. In order to determine the variability from different scanning parameters and scanners accurately, we did not perform any intensity normalization on MR images to prevent another uncertainty on radiomics features or diminishing the effects of various scanning settings.

**Table 8:** The scanning protocols used with the Siemens and Philips 1.5 T MRI scanners.

Scanner		NEX	ST	FOV	Matrix
Siemens	T1	1, 2, 3	2, 3, 4 mm	192, 256, 500	192, 256, 320
	T2	2, 3.4, 4	2, 3, 4 mm	192, 256, 500	N/A
Philips	T1	1, 2, 3	2, 3, 4 mm	192, 256, 512	N/A
	T2	1, 2, 3	2, 3, 4 mm	192, 256, 512	N/A

*NEX* number of excitation, *ST* slice thickness, *FOV* field of view.

### Patient data for the suitability test

First, we investigated the suitability of our phantom materials with brain lesions. A total of 97 patient data identified as having necrosis or progression of brain lesions were used to evaluate the suitability of each phantom material^37^. The use of all patient data were approved and written informed consent was waived by The MD Anderson Cancer Center Institutional Review Board. All MR images of these patients were acquired using a GE 1.5 T MRI scanner with a slice thickness of 5 mm, slice spacing of 6.5 mm, and field-of-view of 22 cm for T1- and T2-weighted images. The brain lesions were segmented on the post-contrast T1 images by a radiation oncologist because the lesions were easier to identify. The post-contrast T1 contour was then rigidly mapped to the other scan sequences such as pre-contrast T1- and T2-weighted images for each patient at each time point using the Velocity AI software (version 3.0.1; Varian Medical Systems, Atlanta, GA, USA).

### Phantom and a healthy volunteer data for the repeatability and variability

For the repeatability and variability of the radiomics features, we used the features from the phantom and a healthy volunteer from two scans. All ROIs on the phantom and a healthy volunteer were delineated semiautomatically using a contour tool available with our in-house imaging software program IBEX^23,38^. Each ROI had a cylindrical shape with a diameter of 1.8 cm and a height of 10 cm for both the phantom and the volunteer. We used axial images where the height is along the z-axis. We used this uniform ROI size on MR images of the phantom and the volunteer to avoid uncertainty between the ROI size and radiomics features. Twenty ROIs on the phantom and volunteer’s brain were delineated (Fig. 4B,C, respectively); Twenty ROIs on a healthy volunteer’s brain were evenly selected over the brain. For patient data, each lesion on MR images for each patient was delineated by ValocityAI software (version 3.0.1; Varian Medical Systems, Atlanta, GA, USA). The radiation oncologist reviewed the contours on the MR images to ensure correct mapping and modified them when necessary.

In this study, we performed image preprocessing before extracting radiomics features to reduce uncertainty in the feature analysis; an edge-preserving smoothing filter was applied to the tumor volume before the feature calculations to preserve meaningful edge information while smoothing out undesirable imaging noise^29^. Then, we extracted a total 76 radiomics features from delineated ROIs from MR images of the phantom, volunteer, and patients, respectively. The radiomics features consisted of 7 Gradient orient histogram features, 22 GLCM features, 11 GLRL features, 31 intensity features, 5 neighborhood gray-tone matrix (NGTDM). The detailed features are listed in Table 9 and Table S1 in the supplementary information. All quantitative image features were calculated and extracted using IBEX^23,38,39^. This software was designed based on MATLAB (version 8.1.0; MathWorks, Natick, MA), and available at http://bit.ly/IBEX_MDAnderson. Our previous study and other work reported volume dependent and gray level dependent features^22,36^. In this study, corrected formulas were used for the volume-dependent features and original formulas were used for the gray level-dependent GLCM features as shown in the Table S2 and S3.

**Table 9:** The examined radiomics features extracted from delineated ROIs on MR images.

Category	Features			
Gradient orient histogram	1. Inter Quartile Range 2. Kurtosis	3. Mean Abs. Deviation 4. Median Abs. Deviation	5. 20 Percentile Area 6. 50 Percentile Area	7. 90 Percentile Area
Gray level co-occurrence Matrix	8. Auto Correlation 9. Cluster Prominence 10. Cluster Shade 11. Cluster tendency 12. Contrast 13. Correlation	14. Difference Entropy 15. Dissimilarity 16. Energy 17. Entropy 18. Homogeneity 19. Homogeneity 2	20. Info. Measure Corr 1 21. Info. Measure Corr 2 22. Inv. Diff Moment Norm 23. Inv. Diff. Norm 24. inverse Variance 25. Max Probability	26. Sum Average 27. Sum entropy 28. Sum Variance 29. Variance
Gray level run length	30. GL Non-uniformity 31. High GL Run Emp 32. Long Run Emp	33. Long Run High GL Emp 34. Long Run Low GL Emp 35. Low GL Run Emp	36. RL Non-uniformity 37. Run Percentage 38. Short Run Emp	39. Short Run High GL Emp 40. Short Run Low GL Emp
Intensity direct	41. Energy 42. Energy Norm 43. Global Entropy 44. Global Mean 45. Global Median 46. Global Std 47. Global Uniformity 48. Interquartile Range	49. Kurtosis 50. Local Entropy Max 51. Local Entropy Mean 52. Local Entropy Median 53. Local Entropy Min 54. Local Entropy std 55. Local Range Mean 56. Local Range Median	57. Local Range Min 58. Local Range Std 59. Local Std Max 60. Local Std Mean 61. Local Std Median 62. Local Std Min 63. Local Std Std 64. Mean Abs. Deviation	65. Median Abs. Deviation 66. 20 Percentile 67. 50 Percentile 68. 90 percentile 69. Root Mean Square 70. Skewness 71. Variance
Neighborhood gray-tone difference	72. Busyness 73. Coarseness	74. Complexity	75. Contrast	76. Texture Strength

### Data analysis

First, we investigated the suitability of each phantom material to see whether the range of radiomics features of each material was similar to the range of radiomics features of the brain lesions of patients. This was done by comparing each feature value from the phantom with those from brain lesions using mean values ± 2 SDs, where this range covers 95% of an approximately normal data set and excludes outliers of the data. This brain lesions of patients only used for the suitability of the phantom materials. Next, we investigated the robustness of the radiomics features obtained from the 20 phantom materials in T1- and T2-weighted images using various scanning protocols and the two scanners. To assess the robustness of the various radiomics features with the different MRI scanning protocol parameters, the COV was computed for each radiomics feature in each scan using Eq. (1)

1 $$\mathrm{COV}=\frac{\sigma }{\mu }\times 100$$

where *σ* is the standard deviation and *μ* is the mean when applying different scanning settings for each MRI parameter (i.e., NEX = 1, 2, and 3).

Next, the repeatability of the radiomics features in two scans was investigated. This was performed with the Siemens 1.5 T MRI scanner twice under the same conditions, such as the same range of whole scanning parameter settings. The repeatability of the radiomics features extracted from normalized images was assessed using the ICC, a measure of the reliability of measurements that can demonstrate how strongly measurements with the same settings resemble each other. For our test–retest scheme with two repeated scans, the ICC was computed using Eq. (2) ^40^

2 $$ICC\left(\mathrm{1,1}\right)= \frac{BMS-WMS}{BMS+WMS}$$

where *BMS* is the between-subjects mean square and *WMS* is the within-subjects mean square. Therefore, the ICC considers the variation in repeated scans in relation to the total variation in the population^40^.

## Supplementary Information

Supplementary Information

## Data Availability

The datasets generated during and/or analyzed during the current study are available from the corresponding author on reasonable request.
