# Supplementary material for: Radiomics feature robustness as measured using an MRI phantom
Source: Sci Rep. 2021 Feb 17;11:3973. doi: 10.1038/s41598-021-83593-3 (PMC7889870; doi:10.1038/s41598-021-83593-3)
Supplement: Supplementary file 1 — Supplementary Information [file 41598_2021_83593_MOESM1_ESM.pdf]

Radiomics feature robustness as measured using an MRI phantom

Joonsang Lee[1,2], Angela Steinmann[1], Yao Ding[1], Hannah Lee[1], Constance Owens[1], Jihong Wang[1], Jinzhong Yang[1], David Followill[1], Rachel Ger[1], Dennis MacKin[1] & Laurence E. Court[1,*]

[1]Department of Radiation Physics, Unit 1420, The University of Texas MD Anderson Cancer Center, Houston, TX 77030, USA.

[2]Department of Computational Medicine and Bioinformatics, University of Michigan, Ann Arbor, MI 48109, USA

*Correspondence and requests for materials should be addressed to L.E.C. (email: lecourt@mdanderson.org)

Table S1. Radiomics features abbreviations and references for the algorithms used in this study

Category	Feature Abbreviation	Feature name	Reference
**Gradient Orient Histogram**	1. InterQuartileRange<br>2. Kurtosis<br>3. MeanAbsDeviation<br>4. MedianAbsDeviation<br>5. 20 PercentileArea<br>6. 50 PercentileArea<br>7. 90 PercentileArea	Inter Quartile Range<br>Kurtosis<br>Mean Absolute Deviation<br>Median Absolute Deviation<br>20 Percentile Area<br>50 Percentile Area<br>90 Percentile Area	Pallavi et al.[1]
**Gray Level Co-occurrence Matrix (GLCM)**	8. AutoCorrelation<br>9. ClusterProminence<br>10. ClusterShade<br>11. Clustertendency<br>12. Contrast<br>13. Correlation<br>14. DifferenceEntropy<br>15. Dissimilarity<br>16. Energy<br>17. Entropy<br>18. Homogeneity<br>19. Homogeneity2<br>20. InfoMeasureCorr1<br>21. InfoMeasureCorr2<br>22. Inv. Diff Moment Norm<br>23. Inv. Diff Norm<br>24. InverseVariance<br>25. MaxProbability<br>26. SumAverage<br>27. SumEntropy<br>28. SumVariance<br>29. Variance	Auto Correlation<br>Cluster Prominence<br>Cluster Shade<br>Cluster tendency<br>Contrast<br>Correlation<br>Difference Entropy<br>Dissimilarity<br>Energy<br>Entropy<br>Homogeneity<br>Homogeneity 2<br>Information Measure Correlation 1<br>Information Measure Correlation 2<br>Inverse Difference Moment Normalized<br>Inverse Difference Normalized<br>Inverse Variance<br>Max Probability<br>Sum Average<br>Sum entropy<br>Sum Variance<br>Variance	Haralick et al.[2]<br>Cunliffe et al.[3]<br>Fried et al.[4]<br>Hunter et al.[5]
**Gray Level Run Length (GLRLM)**	30. GL Non-uniformity<br>31. High GL Run Emp.<br>32. Long Run Emp.<br>33. Long Run High GL Emp.<br>34. Long Run Low GL Emp.<br>35. Low GL Run Emp.<br>36. RL Non-uniformity.<br>37. Run Percentage.<br>38. Short Run Emp.<br>39. Short Run High GL Emp.<br>40. Short Run Low GL Emp.	Gray Level Non-uniformity<br>High Gray Level Run Emphasis<br>Long Run Emphasis<br>Long Run High Gray Level Emphasis<br>Long Run Low Gray Level Emphasis<br>Low Gray Level Run Emphasis<br>Run Length Non-uniformity.<br>Run Percentage.<br>Short Run Emphasis<br>Short Run High Gray Level Run Emphasis<br>Short Run Low Gray Level Run Emphasis	Galloway et al.[6]<br>Tang et al.[7]<br>Hunter et al.[5]
**Intensity direct**	41. Energy<br>42. EnergyNorm<br>43. GlobalEntropy<br>44. GlobalMean<br>45. GlobalMedian<br>46. GlobalStd<br>47. GlobalUniformity<br>48. InterquartileRange<br>49. Kurtosis<br>50. LocalEntropyMax<br>51. LocalEntropyMean<br>52. LocalEntropyMedian<br>53. LocalEntropyMin<br>54. LocalEntropystd<br>55. LocalRangeMean<br>56. LocalRangeMedian<br>57. LocalRangeMin<br>58. LocalRangeStd<br>59. LocalStdMax<br>60. LocalStdMean<br>61. LocalStdMedian<br>62. LocalStdMin<br>63. LocalStdStd<br>64. Mean Abs. Deviation<br>65. Median Abs. Deviation<br>66. 20 Percentile	Energy<br>Energy Norm<br>Global Entropy<br>Global Mean<br>Global Median<br>Global Standard deviation<br>Global Uniformity<br>Interquartile Range<br>Kurtosis<br>Local Entropy Max<br>Local Entropy Mean<br>Local Entropy Median<br>Local Entropy Min<br>Local Entropy Standard deviation<br>Local Range Mean<br>Local Range Median<br>Local Range Min<br>Local Range Standard deviation<br>Local Standard deviation Max<br>Local Standard deviation Mean<br>Local Standard deviation Median<br>Local Standard deviation Min<br>Local Standard deviation Standard deviation<br>Mean Absolute Deviation<br>Median Absolute Deviation<br>20 Percentile	Cunliffe et al.[3]<br>Fried et al.[4]<br>Ganeshan et al.[8-10]<br>Hunter et al.[5]

	67. 50 Percentile<br>68. 90 percentile<br>69. RootMeanSquare<br>70. Skewness<br>71. Variance	50 Percentile<br>90 percentile<br>Root Mean Square<br>Skewness<br>Variance	
**Neighborhood intensity difference**	72. Busyness<br>73. Coarseness<br>74. Complexity<br>75. Contrast<br>76. TextureStrength	Busyness<br>Coarseness<br>Complexity<br>Contrast<br>Texture Strength	Amadasun et al.[11]

Table S2. Formulas for the volume-dependent features that were corrected in IBEX and used in the analysis.

Feature	Original algorithm		Corrected algorithm	
Energy	$\sum_{i}^{N_v} p(i)^2$	[1]	$\frac{1}{N_v}\sum_{i}^{N_v} p(i)^2$	[2]
Gray-level nonuniformity	$\frac{\sum_{i=1}^{N_g}\left[\sum_{j=1}^{N_r} p(i,j)\right]^2}{\sum_{i=1}^{N_g}\sum_{j=1}^{N_r} p(i,j)}$	[3]	$\frac{1}{N_v}\frac{\sum_{i=1}^{N_g}\left[\sum_{j=1}^{N_r} p(i,j)\right]^2}{\sum_{i=1}^{N_g}\sum_{j=1}^{N_r} p(i,j)}$	[4]
Run-length nonuniformity	$\frac{\sum_{i=1}^{N_r}\left[\sum_{j=1}^{N_g} p(i,j)\right]^2}{\sum_{i=1}^{N_g}\sum_{j=1}^{N_r} p(i,j)}$	[5]	$\frac{1}{N_v}\frac{\sum_{i=1}^{N_r}\left[\sum_{j=1}^{N_g} p(i,j)\right]^2}{\sum_{i=1}^{N_g}\sum_{j=1}^{N_r} p(i,j)}$	[6]
Busyness	$\frac{\sum_{i=0}^{G_h} p_i s(i)}{\sum_{i=0}^{G_h}\sum_{j=0}^{G_h} ip_i - jp_i}$	[7]	$\frac{\sum_{i=0}^{G_h} p_i \frac{s(i)}{N(i)}}{\sum_{i=0}^{G_h}\sum_{j=0}^{G_h} ip_i - jp_i}$	[8]
Coarseness	$\left[\epsilon + \sum_{i=0}^{G_h} p_i s(i)\right]^{-1}$	[9]	$\left[\epsilon + \sum_{i=0}^{G_h} p_i \frac{s(i)}{N(i)}\right]^{-1}$	[10]

i: intensity; *N(i)*: number of voxels of each intensity; N_v: the total number of voxels; p_i: probability of intensity in the image; *s(i)*: sum of the average difference value around voxels of intensity; G_h: Highest gray-level intensity; N_g: number of gray levels; N_r: number of run levels; *p(i,j)*: probability of gray-level *i* having a run of length *j*; *X(i)* the intensity of the *i*th voxel in the image.[12,13]

Table S3. Formulas for the gray levels dependent GLCM features

Feature	Original algorithm (F)		Corrected algorithm	
Entropy	$-\sum_{i=1}^{N_g}\sum_{j=1}^{N_g} p(i,j)\ log_2 p(i,j)$	[11]	$\frac{F}{\log[N_g * N_g]}$	[12]
Difference Entropy	$-\sum_{i=0}^{N_g-1} p_{x-y}(i)\ log_2\{p_{x-y}(i)\}$	[13]	$\frac{F}{\log[N_g * N_g]}$	[14]
Sum Entropy	$-\sum_{i=2}^{2N_g} p_{x+y}(i)\ log_2\{p_{x+y}(i)\}$	[15]	$\frac{F}{\log[N_g * N_g]}$	[16]
Contrast	$\sum_{n=0}^{N_g-1} n^2 \left\{\sum_{i=1}^{N_g}\sum_{j=1}^{N_g} p(i,j)\right\}, \quad \lvert i-j\rvert = n$	[17]	$\frac{F}{N_g * N_g}$	[18]
Mean	$\sum_{i=1}^{N_g}\sum_{j=1}^{N_g} p(i,j)$	[19]	$N_g * N_g * F$	[20]
Sum Variance	$\sum_{i=2}^{2N_g} \left\{i + \sum_{i=1}^{N_g}\sum_{j=1}^{N_g} p(i,j)\ log_2[p_{x+y}(i)]\right\}^2 \ log_2\{p_{x+y}(i)\}$	[21]	$\frac{F}{N_g * N_g}$	[22]
Difference Variance	$\sum_{i=2}^{2N_g} \left\{i + \sum_{i=1}^{N_g}\sum_{j=1}^{N_g} p(i,j)\ log_2[p_{x+y}(i)]\right\}^2 \ log_2\{p_{x-y}(i)\}$	[23]	$\frac{F}{N_g * N_g}$	[24]
Sum Average	$\sum_{i=2}^{2N_g} ip_{x+y}(i)$	[25]	$\frac{F}{N_g}$	[26]
Difference Average	$\sum_{i=2}^{2N_g} ip_{x-y}(i)$	[27]	$\frac{F}{N_g}$	[28]
Dissimilarity	$\sum_{i=1}^{N_g}\sum_{j=1}^{N_g} \lvert i-j\rvert\ p(i,j)$	[29]	$\frac{F}{N_g}$	[30]

p(i,j) is the co-occurrence matrix: N_g is the number of discrete gray levels; p_x is the *i*th entry obtained by summing the rows of *p(i,j)*; py is the *j*th entry obtained by summing the column of *p(i,j)* [14]

Table S4. Variations of radiomics features over different MRI scanning settings for T1-weighted images without image reconstruction (small ROI).

**Feature Category**	**COV < 10%**	**10% ≤ COV < 30%**	**COV ≥ 30%**
**Gradient Orient Histogram(7)**	InterQuartileRange<br>Kurtosis, 20Percentile<br>MeanAbsoluteDeviation<br>MedianAbsoluteDeviation<br>50PercentileArea<br>90PercentileArea		
**Gray Level Cooccurence (22)**	Homogeneity<br>Homogeneity2<br>InverseDiffMomentNorm<br>InverseDiffNorm	AutoCorrelation, Contrast<br>Correlation, Energy<br>DifferenceEntropy<br>Entropy, Dissimilarity<br>InformationMeasureCorr1<br>InformationMeasureCorr2<br>InverseVariance<br>MaxProbability, SumVariance<br>SumAverage, SumEntropy	Variance<br>ClusterProminence<br>ClusterShade<br>ClusterTendendcy
**Gray Level Run Length (11)**	GrayLevelNonuniformity<br>ShortRunEmphasis	HighGrayLevelRunEmpha<br>LongRunEmphasis<br>RunLengthNonuniformity<br>RunPercentage	LongRunHighGrayLevelEmpha<br>LongRunLowGrayLevelEmpha<br>LowGrayLevelRunEmpha<br>ShortRunHighGrayLevelEmpha<br>ShortRunLowGrayLevelEmpha
**Intensity Direct (31)**	LocalEntropyMax	EnergyNorm, GlobalEntropy<br>GlobalMean<br>GlobalMedian, GlobalStd<br>GlobalUniformity<br>InterQuartileRange<br>Kurtosis, LocalEntropyMean<br>LocalEntropyMedian<br>LocalEntropyStd<br>LocalRangeMean<br>LocalRangeMedian<br>LocalRangeMin,<br>LocalRangeStd<br>LocalStdMax, LocalStdMean<br>LocalStdMedian, LocalStdMin<br>LocalStdStd<br>MeanAbsoluteDeviation<br>MedianAbsoluteDeviation<br>20Percentile, 50Percentile<br>90Percentile,<br>RootMeanSquare	Energy<br>LocalEntropyMin<br>Skewness<br>Variance
**Neighbor Intensity Difference (5)**		Coarseness	Busyness, Complexity<br>Contrast<br>TextureStrength
**Average COV**	4.59%	17.80%	45.73%

Table S5. Variations of radiomics features over different MRI scanning settings for T1-weighted images with normalization.

Feature Category	COV < 10%	10% ≤ COV < 30%	COV ≥ 30%
**Gradient Orient Histogram (7)**	InterQuartileRange<br>Kurtosis<br>MeanAbsoluteDeviation<br>MedianAbsoluteDeviation<br>20Percentile<br>50PercentileArea	90PercentileArea	
**Gray Level Cooccurence (22)**	Homogeneity<br>Homogeneity2<br>InformationMeasureCorr2<br>InverseDiffMomentNorm<br>InverseDiffMomentNorm<br>InverseVariance	AutoCorrelation<br>Contrast, Correlation<br>DifferenceEntropy<br>Dissimilarity, Entropy<br>InformationMeasureCorr1<br>MaxProbability<br>SumAverage, SumEntropy<br>SumVariance	ClusterProminence<br>ClusterShade<br>ClusterTendendcy<br>Energy<br>Variance
**Gray Level Run Length (11)**	GrayLevelNonuniformity<br>ShortRunEmphasis	HighGrayLevelRunEmpha<br>RunLengthNonuniformity<br>RunPercentage<br>ShortRunEmphasis	LongRunEmphasis<br>LongRunHighGrayLevelEmpha<br>LongRunLowGrayLevelEmpha<br>LowGrayLevelRunEmpha<br>ShortRunLowGrayLevelEmpha
**Intensity Direct (31)**	LocalEntropyMax	Energy, EnergyNorm<br>GlobalMean, GlobalStd<br>GlobalEntropy, GlobalMedian<br>GlobalUniformity<br>InterQuartileRange<br>LocalEntropyMean<br>LocalEntropyMedian<br>LocalEntropyMin, LocalRangeMean<br>LocalRangeMin<br>LocalRangeStd, LocalStdMax<br>LocalStdMean, LocalRangeMedian<br>LocalStdMin, LocalStdStd<br>LocalEntropyStd, LocalStdMedian<br>MeanAbsoluteDeviation<br>MedianAbsoluteDeviation<br>20Percentile, 50Percentile<br>90Percentile<br>RootMeanSquare	Skewness<br>Variance<br>Kurtosis
**Neighbor Intensity Difference (5)**		Coarseness	Busyness<br>Complexity<br>Contrast<br>TextureStrength
**Average COV**	4.87%	16.83%	48.32%

Table S6. Variations of radiomics features over different MRI scanning settings for T1-weighted images with smoothing filter.

Feature Category	COV < 10%	10% ≤ COV < 30%	COV ≥ 30%
**Gradient Orient Histogram**	InterQuartileRange<br>Kurtosis<br>MeanAbsoluteDeviation<br>MedianAbsoluteDeviation<br>50PercentileArea<br>90PercentileArea	20Percentile	
**Gray Level Cooccurence**	Correlation<br>Homogeneity<br>Homogeneity2<br>InformationMeasureCorr1<br>InformationMeasureCorr2<br>InverseDiffMomentNorm<br>InverseDiffNorm	AutoCorrelation<br>InverseVariance<br>Contrast, Energy<br>DifferenceEntropy<br>Dissimilarity, Entropy<br>MaxProbability<br>SumAverage<br>SumEntropy	SumVariance<br>Variance<br>ClusterProminence<br>ClusterShade<br>ClusterTendendcy
**Gray Level Run Length**	GrayLevelNonuniformity<br>ShortRunEmphasis	HighGrayLevelRunEmpha<br>LongRunEmphasis<br>RunLengthNonuniformity<br>RunPercentage	LongRunHighGrayLevelEmpha<br>LongRunLowGrayLevelEmpha<br>LowGrayLevelRunEmpha<br>ShortRunHighGrayLevelEmpha<br>ShortRunLowGrayLevelEmpha
**Intensity Direct**	LocalEntropyMax	GlobalEntropy<br>GlobalMean<br>GlobalMedian, GlobalStd<br>GlobalUniformity<br>InterQuartileRange<br>LocalEntropyMean<br>LocalEntropyMedian<br>LocalEntropyStd<br>LocalRangeMean<br>LocalRangeMedian<br>LocalRangeMin, LocalRangeStd<br>LocalStdMax,LocalStdMean<br>LocalStdMedian<br>LocalStdMin, LocalStdStd<br>MeanAbsoluteDeviation<br>MedianAbsoluteDeviation<br>20Percentile, 50Percentile<br>90Percentile<br>Kurtosis<br>RootMeanSquare	Energy<br>EnergyNorm<br>LocalEntropyMin<br>Skewness<br>Variance
**Neighbor Intensity Difference**		Coarseness	Busyness<br>Complexity<br>Contrast<br>TextureStrength
**Average COV**	4.29%	17.72%	45.09%

Table S7. Variations of radiomics features over different MRI scanning settings for T2-weighted images without image reconstruction (small ROI).

Feature Category	COV < 10%	10% ≤ COV < 30%	COV ≥ 30%
**Gradient Orient Histogram**	InterQuartileRange<br>Kurtosis<br>MeanAbsoluteDeviation<br>MedianAbsoluteDeviation<br>20Percentile<br>50PercentileArea<br>90PercentileArea		
**Gray Level Cooccurence**	DifferenceEntropy<br>Entropy<br>Homogeneity<br>Homogeneity2<br>InformationMeasureCorr2<br>InverseDiffNorm<br>InverseVariance<br>MaxProbability<br>SumEntropy<br>InverseDiffMomentNorm	Contrast<br>Correlation<br>Dissimilarity<br>Energy<br>SumAverage<br>InformationMeasureCorr1	AutoCorrelation<br>ClusterProminence<br>ClusterShade<br>ClusterTendendcy<br>SumVariance<br>Variance
**Gray Level Run Length**	GrayLevelNonuniformity<br>RunPercentage<br>ShortRunEmphasis	LongRunEmphasis<br>LongRunHighGrayLevelEmpha<br>LongRunLowGrayLevelEmpha<br>LowGrayLevelRunEmpha<br>RunLengthNonuniformity<br>ShortRunLowGrayLevelEmpha	HighGrayLevelRunEmpha<br>ShortRunHighGrayLevelEmpha
**Intensity Direct**	GlobalEntropy<br>GlobalUniformity<br>LocalEntropyMax<br>LocalEntropyMean<br>LocalEntropyMedian<br>LocalEntropyStd	GlobalMean<br>GlobalMedian, GlobalStd<br>InterQuartileRange<br>LocalEntropyMin<br>LocalRangeMean, LocalRangeMedian<br>LocalRangeMin, LocalStdMax<br>LocalStdMean, LocalRangeStd<br>LocalStdMedian<br>LocalStdMin, LocalStdStd<br>MeanAbsoluteDeviation<br>MedianAbsoluteDeviation<br>20Percentile, 50Percentile<br>90Percentile<br>Kurtosis<br>RootMeanSquare	Energy<br>EnergyNorm<br>Skewness<br>Variance
**Neighbor Intensity Difference**		Coarseness<br>Contrast	Busyness<br>Complexity<br>TextureStrength
**Average COV**	5.67%	17.41%	45.68%

Table S8. Variations of radiomics features over different MRI scanning settings for T2-weighted images with normalization.

Feature Category	COV < 10%	10% ≤ COV < 30%	COV ≥ 30%
**Gradient Orient Histogram**	InterQuartileRange<br>Kurtosis<br>MeanAbsoluteDeviation<br>MedianAbsoluteDeviation<br>50PercentileArea<br>90PercentileArea	20Percentile	
**Gray Level Cooccurence**	Homogeneity<br>Homogeneity2<br>InverseDiffMomentNorm<br>InverseDiffNorm	Correlation<br>DifferenceEntropy<br>Dissimilarity<br>Energy<br>Entropy<br>InformationMeasureCorr1<br>InformationMeasureCorr2<br>InverseVariance<br>MaxProbability<br>SumAverage<br>SumEntropy	AutoCorrelation<br>ClusterProminence<br>ClusterShade<br>ClusterTendendcy<br>Contrast<br>SumVariance<br>Variance
**Gray Level Run Length**	ShortRunEmphasis	GrayLevelNonuniformity<br>LongRunEmphasis<br>LongRunLowGrayLevelEmpha<br>LowGrayLevelRunEmpha<br>RunLengthNonuniformity<br>RunPercentage<br>ShortRunLowGrayLevelEmpha	HighGrayLevelRunEmpha<br>LongRunHighGrayLevelEmpha<br>ShortRunHighGrayLevelEmpha
**Intensity Direct**	LocalEntropyMax<br>LocalEntropyStd<br>LocalStdMax	GlobalEntropy<br>GlobalStd<br>GlobalUniformity<br>InterQuartileRange<br>LocalEntropyMean<br>LocalEntropyMedian<br>LocalRangeMean<br>LocalRangeMedian<br>LocalRangeMin<br>LocalRangeStd<br>LocalStdMean<br>LocalStdMedian<br>LocalStdMin<br>MeanAbsoluteDeviation<br>MedianAbsoluteDeviation<br>LocalStdStd<br>Kurtosis	Energy<br>EnergyNorm<br>GlobalMean<br>GlobalMedian<br>LocalEntropyMin<br>20Percentile<br>50Percentile<br>90Percentile<br>RootMeanSquare<br>Skewness<br>Variance
**Neighbor Intensity Difference**		Busyness<br>Coarseness<br>Contrast	Complexity<br>TextureStrength
**Average COV**	4.67%	19.35%	45.92%

Table S9. Variations of radiomics features over different MRI scanning settings for T2-weighted images with smoothing filter.

Feature Category	COV < 10%	10% ≤ COV < 30%	COV ≥ 30%
**Gradient Orient Histogram**	InterQuartileRange<br>Kurtosis<br>MeanAbsoluteDeviation<br>MedianAbsoluteDeviation<br>50PercentileArea<br>90PercentileArea	20Percentile	
**Gray Level Cooccurence**	Correlation<br>Homogeneity<br>Homogeneity2<br>InformationMeasureCorr1<br>InformationMeasureCorr2<br>InverseDiffMomentNorm<br>InverseDiffNorm<br>MaxProbability	DifferenceEntropy<br>Dissimilarity<br>Energy<br>Entropy<br>InverseVariance<br>SumAverage<br>SumEntropy	ClusterProminence<br>ClusterShade<br>ClusterTendendcy<br>Contrast<br>SumVariance<br>Variance<br>AutoCorrelation
**Gray Level Run Length**	GrayLevelNonuniformity<br>ShortRunEmphasis	LongRunEmphasis<br>LongRunLowGrayLevelEmpha<br>LowGrayLevelRunEmpha<br>RunLengthNonuniformity<br>RunPercentage<br>ShortRunLowGrayLevelEmpha	HighGrayLevelRunEmpha<br>LongRunHighGrayLevelEmpha<br>ShortRunHighGrayLevelEmpha
**Intensity Direct**	GlobalUniformity<br>LocalEntropyMax<br>LocalEntropyStd	GlobalEntropy<br>GlobalStd<br>InterQuartileRange<br>LocalEntropyMean<br>LocalEntropyMedian<br>LocalRangeMean<br>LocalRangeMedian<br>LocalRangeMin<br>LocalRangeStd<br>LocalStdMax<br>LocalStdMean<br>LocalStdMedian<br>LocalStdMin<br>LocalStdStd<br>MeanAbsoluteDeviation<br>MedianAbsoluteDeviation<br>Kurtosis	Energy<br>EnergyNorm<br>GlobalMean<br>GlobalMedian<br>LocalEntropyMin<br>20Percentile<br>50Percentile<br>90Percentile<br>RootMeanSquare<br>Skewness<br>Variance
**Neighbor Intensity Difference**		Busyness<br>Coarseness<br>Contrast	Complexity<br>TextureStrength
**Average COV**	4.69%	19.66%	52.16%

Table S10. The feature differences between non-normalization and normalization in small ROIs

Feature category	T1	T2
Gradient Orient Histogram	90PercentileArea	N/A
Gray level Cooccurence	DifferenceEntropy, InformationMeasureCorr1, InformationMeasureCorr2, InverseVariance, SumVariance, SumEntropy	Entropy, Contrast, MaxProbability
Gray level Run Length	N/A	N/A
Intensity	EnergyNorm, GlobalEntropy, localEntropyMean	GlobalEntropy, LocalEntropyMedian, GlobalMean, LocalStdMax, 20Percentile, 50Percentile, 90Percentile, RootMeanSquare, Skewness
Neighbor Intensity Difference	N/A	Busyness
Average COV	22.15% vs 21.25%	23.24% vs 18.97%

Table S11. The feature differences between non-normalization and normalization

Feature category	T1	T2
Gradient Orient Histogram	N/A	20Percentile
Gray level Cooccurence	DifferenceEntropy, SumEntropy, Energy, SumVariance	DifferenceEntropy, nformationMeasureCorr2, SumEntropy, InverseVariance
Gray level Run Length	N/A	GrayLevelNonuniformity, RunPercentage
Intensity	GlobalEntropy, LocalEntropyMean, Kurtosis, Energy, EnergyNorm, LocalEntropyMin	GlobalUniformity, LocalEntropyMean, Skewness
Neighbor Intensity Difference	N/A	N/A
Average COV	22.15% VS 22.30%	23.24% vs 24.69%

Table S12. The feature differences between non-smoothing and smoothing filter (Butterworth)

Feature category	T1	T2
Gradient Orient Histogram	20Percentile, 90PercentileArea	20Percentile
Gray level Cooccurence	InverseVariance, SumEntropy, Correlation, InformationMeasureCorr1	DifferenceEntropy, SumEntropy, InverseVariance, Correlation, InformationMeasureCorr1 MaxProbability
Gray level Run Length	N/A	RunPercentage
Intensity	GlobalEntropy, LocalEntropyMean	LocalEntropyMean, LocalStdMax, Skewness
Neighbor Intensity Difference	N/A	N/ A
Average COV	22.15% VS 21.74%	23.24% vs 25.75%

Table S13. The full feature names for the cluster heat maps of the variability of radiomics features with different MRI scanning settings for T1-weighted images (Fig. 2 (a)) and T2-weighted images (Fig. 2 (b)).

Top to bottom	**T1-weighted images**	**T2-weighted images**
1	F2-ClusterProminence	F2-ClusterProminence
2	F2-ClusterShade	F2-ClusterShade
3	F4-LocalEntropyMin	F4-InterQuartileRange
4	F1-50PercentileArea	F4-MeanAbsoluteDeviation
5	F1-InterQuartileRange	F4-MedianAbsoluteDeviation
6	F1-MedianAbsoluteDeviation	F4-GlobalStd
7	F1-Kurtosis	F4-LocalRangeMedian
8	F1-MeanAbsoluteDeviation	F4-LocalStdMedian
9	F1-90PercentileArea	F3-RunLengthNonuniformity
10	F2-InverseDiffMomentNorm	F4-LocalRangeMean
11	F2-InverseDiffNorm	F4-LocalStdMean
12	F4-LocalEntropyMax	F2-Dissimilarity
13	F2-Homogeneity	F4-LocalRangeMin
14	F2-Homogeneity2	F4-LocalStdMin
15	F1-20Percentile	F5-Contrast
16	F2-InverseVariance	F3-LongRunLowGrayLevelEmpha
17	F2-Correlation	F5-Busyness
18	F2-InformationMeasureCorr2	F3-LongRunEmphasis
19	F3-ShortRunEmphasis	F4-Kurtosis
20	F4-LocalEntropyMedian	F1-90PercentileArea
21	F3-RunPercentage	F2-InverseDiffMomentNorm
22	F3-GrayLevelNonuniformity	F2-InverseDiffNorm
23	F4-LocalEntropyMean	F4-LocalEntropyMax
24	F2-Entropy	F3-ShortRunEmphasis
25	F2-DifferenceEntropy	F2-Homogeneity
26	F2-SumEntropy	F2-Homogeneity2
27	F4-GlobalEntropy	F1-Kurtosis
28	F2-InformationMeasureCorr1	F1-MeanAbsoluteDeviation
29	F4-LocalEntropyStd	F1-50PercentileArea
30	F4-20Percentile	F1-InterQuartileRange
31	F4-RootMeanSquare	F1-MedianAbsoluteDeviation
32	F4-GlobalMean	F4-LocalStdMax
33	F4-GlobalMedian	F3-GrayLevelNonuniformity
34	F4-50Percentile	F1-20Percentile
35	F2-SumAverage	F4-LocalEntropyStd
36	F4-90Percentile	F2-Correlation
37	F3-RunLengthNonuniformity	F2-Entropy
38	F4-LocalRangeMin	F2-InformationMeasureCorr2
39	F4-LocalStdMin	F2-InverseVariance
40	F5-Coarseness	F4-LocalEntropyMean
41	F2-MaxProbability	F4-LocalEntropyMedian
42	F4-GlobalUniformity	F2-DifferenceEntropy
43	F2-Energy	F3-RunPercentage
44	F4-MedianAbsoluteDeviation	F2-SumEntropy
45	F4-InterQuartileRange	F4-GlobalEntropy
46	F4-LocalRangeMedian	F2-MaxProbability

47	F4-LocalStdMedian	F4-GlobalUniformity
48	F4-LocalRangeStd	F2-InformationMeasureCorr1
49	F4-LocalStdStd	F2-Energy
50	F2-Dissimilarity	F5-Coarseness
51	F4-LocalStdMax	F3-LowGrayLevelRunEmpha
52	F4-LocalRangeMean	F3-ShortRunLowGrayLevelEmpha
53	F4-LocalStdMean	F4-LocalRangeStd
54	F4-GlobalStd	F4-LocalStdStd
55	F4-MeanAbsoluteDeviation	F4-LocalEntropyMin
56	F4-Energy	F4-Variance
57	F3-LongRunHighGrayLevelEmpha	F2-ClusterTendendcy
58	F5-TextureStrength	F2-Variance
59	F5-Contrast	F4-Energy
60	F3-LongRunEmphasis	F2-SumVariance
61	F4-Kurtosis	F4-EnergyNorm
62	F4-Variance	F5-Complexity
63	F2-ClusterTendendcy	F5-TextureStrength
64	F2-Variance	F2-AutoCorrelation
65	F2-Contrast	F3-HighGrayLevelRunEmpha
66	F2-SumVariance	F3-ShortRunHighGrayLevelEmpha
67	F3-ShortRunHighGrayLevelEmpha	F4-GlobalMean
68	F4-EnergyNorm	F4-RootMeanSquare
69	F2-AutoCorrelation	F4-20Percentile
70	F3-HighGrayLevelRunEmpha	F4-GlobalMedian
71	F5-Complexity	F4-50Percentile
72	F4-Skewness	F2-SumAverage
73	F3-LongRunLowGrayLevelEmpha	F2-Contrast
74	F5-Busyness	F4-90Percentile
75	F3-LowGrayLevelRunEmpha	F3-LongRunHighGrayLevelEmpha
76	F3-ShortRunLowGrayLevelEmpha	F4-Skewnes

F1: Gradient Orient Histogram
F2: Gray Level Cooccurence
F3: Gray Level Run Length
F4: Intensity Direct
F5: Neighbor Intensity Difference

Table S14. The full feature names for the cluster heat maps for T1-weighted images (Fig. 2 (c)) and T2-weighted images (Fig. 2 (d)) with normalization.

Top to bottom	**T1-weighted images**	**T2-weighted images**
1	F4-Energy	F2-ClusterShade
2	F2-SumVariance	F4-LocalEntropyMin
3	F3-ShortRunHighGrayLevelEmpha	F4-Skewness
4	F4-EnergyNorm	F2-ClusterProminence
5	F2-AutoCorrelation	F4-GlobalMean
6	F3-HighGrayLevelRunEmpha	F4-RootMeanSquare
7	F3-RunLengthNonuniformity	F4-20Percentile
8	F4-GlobalStd	F4-GlobalMedian
9	F4-MeanAbsoluteDeviation	F4-50Percentile
10	F2-MaxProbability	F5-TextureStrength
11	F5-Coarseness	F3-HighGrayLevelRunEmpha
12	F2-Contrast	F2-AutoCorrelation
13	F4-LocalRangeMin	F3-ShortRunHighGrayLevelEmpha
14	F4-LocalStdMin	F2-SumVariance
15	F4-LocalRangeMedian	F4-EnergyNorm
16	F4-LocalStdMedian	F4-Energy
17	F4-InterQuartileRange	F5-Complexity
18	F4-MedianAbsoluteDeviation	F3-LowGrayLevelRunEmpha
19	F2-InformationMeasureCorr1	F3-ShortRunLowGrayLevelEmpha
20	F2-Dissimilarity	F2-InformationMeasureCorr2
21	F4-GlobalUniformity	F3-GrayLevelNonuniformity
22	F1-20Percentile	F3-ShortRunEmphasis
23	F1-90PercentileArea	F3-RunPercentage
24	F2-InverseDiffMomentNorm	F2-DifferenceEntropy
25	F2-InverseDiffNorm	F4-GlobalEntropy
26	F1-Kurtosis	F4-GlobalStd
27	F1-MeanAbsoluteDeviation	F2-SumEntropy
28	F1-50PercentileArea	F2-Entropy
29	F1-InterQuartileRange	F2-InverseVariance
30	F1-MedianAbsoluteDeviation	F2-InformationMeasureCorr1
31	F3-ShortRunEmphasis	F4-LocalRangeMean
32	F4-LocalEntropyMax	F4-LocalStdMean
33	F2-Homogeneity	F2-Correlation
34	F2-Homogeneity2	F4-LocalEntropyMean
35	F2-InverseVariance	F4-LocalEntropyMedian
36	F2-InformationMeasureCorr2	F4-LocalEntropyStd
37	F3-GrayLevelNonuniformity	F1-20Percentile
38	F4-LocalStdMax	F2-MaxProbability
39	F4-LocalRangeStd	F4-GlobalUniformity
40	F4-LocalStdStd	F4-LocalStdMax
41	F2-Correlation	F4-LocalRangeStd
42	F4-LocalEntropyStd	F4-LocalStdStd
43	F4-20Percentile	F1-90PercentileArea
44	F4-LocalRangeMean	F2-InverseDiffMomentNorm
45	F4-LocalStdMean	F2-InverseDiffNorm
46	F4-RootMeanSquare	F1-50PercentileArea
47	F4-GlobalMean	F1-InterQuartileRange

48	F4-GlobalMedian	F1-MedianAbsoluteDeviation
49	F4-50Percentile	F4-LocalEntropyMax
50	F2-SumAverage	F1-Kurtosis
51	F4-90Percentile	F1-MeanAbsoluteDeviation
52	F2-Entropy	F2-Homogeneity
53	F2-DifferenceEntropy	F2-Homogeneity2
54	F2-SumEntropy	F3-LongRunHighGrayLevelEmpha
55	F4-GlobalEntropy	F4-LocalRangeMin
56	F4-LocalEntropyMean	F4-LocalStdMin
57	F3-RunPercentage	F3-LongRunEmphasis
58	F4-LocalEntropyMedian	F2-Energy
59	F4-LocalEntropyMin	F5-Coarseness
60	F5-Complexity	F3-LongRunLowGrayLevelEmpha
61	F4-Variance	F4-Kurtosis
62	F2-ClusterTendendcy	F4-MeanAbsoluteDeviation
63	F2-Variance	F5-Busyness
64	F5-TextureStrength	F5-Contrast
65	F4-Kurtosis	F4-InterQuartileRange
66	F2-Energy	F4-MedianAbsoluteDeviation
67	F5-Contrast	F4-LocalRangeMedian
68	F3-LongRunEmphasis	F4-LocalStdMedian
69	F3-LongRunHighGrayLevelEmpha	F2-SumAverage
70	F4-Skewness	F4-90Percentile
71	F3-ShortRunLowGrayLevelEmpha	F4-Variance
72	F3-LowGrayLevelRunEmpha	F2-ClusterTendendcy
73	F5-Busyness	F2-Variance
74	F3-LongRunLowGrayLevelEmpha	F2-Contrast
75	F2-ClusterProminence	F2-Dissimilarity
76	F2-ClusterShade	F3-RunLengthNonuniformity

F1: Gradient Orient Histogram
F2: Gray Level Cooccurence
F3: Gray Level Run Length
F4: Intensity Direct
F5: Neighbor Intensity Difference

Table S15. The full feature names for the cluster heat maps for T1-weighted images (Fig. 2 (c)) and T2-weighted images (Fig. 2 (d)) with a smoothing filter.

Top to bottom	**T1-weighted images**	**T2-weighted images**
1	F2-InformationMeasureCorr1	F2-ClusterProminence
2	F3-GrayLevelNonuniformity	F2-ClusterShade
3	F4-LocalEntropyMax	F1-90PercentileArea
4	F2-Correlation	F2-InverseDiffMomentNorm
5	F2-InformationMeasureCorr2	F2-InverseDiffNorm
6	F1-Kurtosis	F2-Homogeneity
7	F1-MeanAbsoluteDeviation	F2-Homogeneity2
8	F1-50PercentileArea	F1-Kurtosis
9	F1-InterQuartileRange	F1-MeanAbsoluteDeviation
10	F1-MedianAbsoluteDeviation	F3-ShortRunEmphasis
11	F2-Homogeneity	F2-MaxProbability
12	F2-Homogeneity2	F4-GlobalUniformity
13	F1-90PercentileArea	F2-InformationMeasureCorr2
14	F2-InverseDiffMomentNorm	F2-Correlation
15	F2-InverseDiffNorm	F4-LocalEntropyMax
16	F2-MaxProbability	F2-InformationMeasureCorr1
17	F4-GlobalUniformity	F3-GrayLevelNonuniformity
18	F3-ShortRunEmphasis	F4-LocalEntropyStd
19	F2-Entropy	F1-50PercentileArea
20	F3-RunPercentage	F1-InterQuartileRange
21	F2-SumEntropy	F1-MedianAbsoluteDeviation
22	F4-LocalEntropyMedian	F4-GlobalEntropy
23	F2-DifferenceEntropy	F2-Entropy
24	F4-GlobalEntropy	F2-DifferenceEntropy
25	F4-LocalEntropyMean	F2-InverseVariance
26	F2-InverseVariance	F2-SumEntropy
27	F4-LocalEntropyStd	F4-LocalEntropyMedian
28	F1-20Percentile	F3-RunPercentage
29	F4-Kurtosis	F4-LocalEntropyMean
30	F4-LocalRangeMin	F3-LowGrayLevelRunEmpha
31	F4-LocalStdMin	F3-ShortRunLowGrayLevelEmpha
32	F3-LongRunEmphasis	F4-LocalStdMax
33	F3-RunLengthNonuniformity	F2-Energy
34	F2-SumAverage	F5-Coarseness
35	F4-20Percentile	F4-LocalRangeStd
36	F4-90Percentile	F4-LocalStdStd
37	F4-GlobalMedian	F3-LongRunLowGrayLevelEmpha
38	F4-50Percentile	F3-LongRunEmphasis
39	F4-GlobalMean	F4-Kurtosis
40	F4-RootMeanSquare	F1-20Percentile
41	F4-MedianAbsoluteDeviation	F5-Contrast
42	F4-InterQuartileRange	F4-LocalRangeMean
43	F4-LocalRangeMedian	F4-LocalStdMean
44	F4-LocalStdMedian	F2-Dissimilarity
45	F2-Energy	F3-RunLengthNonuniformity
46	F2-Dissimilarity	F5-Busyness
47	F4-LocalRangeMean	F4-MedianAbsoluteDeviation

48	F4-LocalStdMean	F4-LocalRangeMin
49	F4-GlobalStd	F4-LocalStdMin
50	F4-MeanAbsoluteDeviation	F4-LocalRangeMedian
51	F5-Coarseness	F4-LocalStdMedian
52	F4-LocalStdMax	F4-InterQuartileRange
53	F4-LocalRangeStd	F4-GlobalStd
54	F4-LocalStdStd	F4-MeanAbsoluteDeviation
55	F5-Complexity	F2-SumAverage
56	F5-TextureStrength	F3-LongRunHighGrayLevelEmpha
57	F3-LongRunLowGrayLevelEmpha	F4-20Percentile
58	F3-LowGrayLevelRunEmpha	F4-GlobalMedian
59	F3-ShortRunLowGrayLevelEmpha	F4-50Percentile
60	F5-Busyness	F4-GlobalMean
61	F4-Variance	F4-RootMeanSquare
62	F2-ClusterTendendcy	F2-Contrast
63	F2-Variance	F4-90Percentile
64	F3-LongRunHighGrayLevelEmpha	F4-LocalEntropyMin
65	F4-Skewness	F4-Skewness
66	F4-Energy	F4-Variance
67	F2-AutoCorrelation	F2-ClusterTendendcy
68	F3-HighGrayLevelRunEmpha	F2-Variance
69	F2-SumVariance	F2-AutoCorrelation
70	F3-ShortRunHighGrayLevelEmpha	F3-HighGrayLevelRunEmpha
71	F4-EnergyNorm	F5-Complexity
72	F2-Contrast	F5-TextureStrength
73	F5-Contrast	F4-Energy
74	F4-LocalEntropyMin	F3-ShortRunHighGrayLevelEmpha
75	F2-ClusterProminence	F2-SumVariance
76	F2-ClusterShade	F4-EnergyNorm

F1: Gradient Orient Histogram
F2: Gray Level Cooccurence
F3: Gray Level Run Length
F4: Intensity Direct
F5: Neighbor Intensity Difference

Table S16. The full feature names for the cluster heat maps for T1-weighted images (Fig. 2 (c)) and T2-weighted images (Fig. 2 (d)) with small ROIs (diameter of 1.2 cm).

Top to bottom	**T1-weighted images**	**T2-weighted images**
1	F2-ClusterProminence	F2-ClusterProminence
2	F2-ClusterShade	F2-ClusterShade
3	F5-Complexity	F1-90PercentileArea
4	F4-Skewness	F2-InverseDiffMomentNorm
5	F3-LongRunLowGrayLevelEmpha	F2-InverseDiffNorm
6	F5-Busyness	F1-InterQuartileRange
7	F3-LowGrayLevelRunEmpha	F1-MedianAbsoluteDeviation
8	F3-ShortRunLowGrayLevelEmpha	F1-50PercentileArea
9	F4-LocalEntropyMin	F2-Homogeneity
10	F1-50PercentileArea	F2-Homogeneity2
11	F1-InterQuartileRange	F1-Kurtosis
12	F1-MedianAbsoluteDeviation	F1-MeanAbsoluteDeviation
13	F1-Kurtosis	F2-MaxProbability
14	F1-MeanAbsoluteDeviation	F4-LocalEntropyStd
15	F2-Homogeneity	F1-20Percentile
16	F2-Homogeneity2	F4-GlobalUniformity
17	F1-90PercentileArea	F4-GlobalEntropy
18	F2-InverseDiffMomentNorm	F4-LocalEntropyMean
19	F2-InverseDiffNorm	F4-LocalEntropyMedian
20	F1-20Percentile	F2-InformationMeasureCorr2
21	F2-Entropy	F2-InverseVariance
22	F2-DifferenceEntropy	F2-Entropy
23	F2-SumEntropy	F2-DifferenceEntropy
24	F3-RunPercentage	F2-SumEntropy
25	F4-LocalEntropyMedian	F3-GrayLevelNonuniformity
26	F4-GlobalEntropy	F3-RunPercentage
27	F4-LocalEntropyMean	F3-ShortRunEmphasis
28	F4-LocalEntropyMax	F4-LocalEntropyMax
29	F3-GrayLevelNonuniformity	F3-LongRunLowGrayLevelEmpha
30	F3-ShortRunEmphasis	F3-LowGrayLevelRunEmpha
31	F4-LocalEntropyStd	F3-ShortRunLowGrayLevelEmpha
32	F2-Correlation	F2-Correlation
33	F2-InformationMeasureCorr2	F4-Kurtosis
34	F2-InverseVariance	F3-LongRunEmphasis
35	F2-InformationMeasureCorr1	F2-Energy
36	F4-20Percentile	F5-Coarseness
37	F4-RootMeanSquare	F4-LocalStdMax
38	F4-GlobalMean	F4-LocalRangeStd
39	F4-GlobalMedian	F4-LocalStdStd
40	F4-50Percentile	F4-GlobalStd
41	F2-SumAverage	F4-MedianAbsoluteDeviation
42	F4-90Percentile	F4-LocalRangeMedian
43	F4-LocalRangeMin	F4-LocalStdMedian
44	F4-LocalStdMin	F2-InformationMeasureCorr1
45	F5-Coarseness	F3-RunLengthNonuniformity
46	F2-MaxProbability	F4-LocalRangeMin
47	F4-GlobalUniformity	F4-LocalStdMin

48	F3-RunLengthNonuniformity	F2-Dissimilarity
49	F4-InterQuartileRange	F4-LocalRangeMean
50	F4-MedianAbsoluteDeviation	F4-LocalStdMean
51	F4-LocalRangeStd	F2-Contrast
52	F4-LocalStdStd	F4-LocalEntropyMin
53	F2-Energy	F5-Contrast
54	F2-Dissimilarity	F4-InterQuartileRange
55	F4-LocalRangeMean	F4-MeanAbsoluteDeviation
56	F4-LocalStdMean	F2-SumAverage
57	F4-LocalRangeMedian	F4-20Percentile
58	F4-LocalStdMedian	F4-GlobalMedian
59	F4-MeanAbsoluteDeviation	F4-50Percentile
60	F4-GlobalStd	F3-LongRunHighGrayLevelEmpha
61	F4-LocalStdMax	F4-90Percentile
62	F5-TextureStrength	F4-GlobalMean
63	F4-Variance	F4-RootMeanSquare
64	F2-ClusterTendendcy	F4-Variance
65	F2-Variance	F2-ClusterTendendcy
66	F3-LongRunHighGrayLevelEmpha	F2-Variance
67	F4-Energy	F4-Energy
68	F2-Contrast	F2-SumVariance
69	F4-EnergyNorm	F4-EnergyNorm
70	F2-AutoCorrelation	F5-TextureStrength
71	F3-HighGrayLevelRunEmpha	F5-Complexity
72	F2-SumVariance	F3-ShortRunHighGrayLevelEmpha
73	F3-ShortRunHighGrayLevelEmpha	F2-AutoCorrelation
74	F3-LongRunEmphasis	F3-HighGrayLevelRunEmpha
75	F4-Kurtosis	F4-Skewness
76	F5-Contrast	F5-Busyness

F1: Gradient Orient Histogram
F2: Gray Level Cooccurence
F3: Gray Level Run Length
F4: Intensity Direct
F5: Neighbor Intensity Difference

Supplemental Document Reference

1 Pallavi, T. *et al.* Texture Descriptors to distinguish Radiation Necrosis from Recurrent Brain Tumors on multi-parametric MRI. *Proc SPIE Int Soc Opt Eng* **9035**, 90352B, doi:10.1117/12.2043969 (2014).

2 Haralick, R. M., Shanmugam, K. & Dinstein, I. Textural Features for Image Classification. *Ieee T Syst Man Cyb* **Smc3**, 610-621, doi:Doi 10.1109/Tsmc.1973.4309314 (1973).

3 Cunliffe, A. R., Armato, S. G., Fei, X. H. M., Tuohy, R. E. & Al-Hallaq, H. A. Lung texture in serial thoracic CT scans: Registration- based methods to compare anatomically matched regions. *Medical Physics* **40**, doi:Artn 06190610.1118/1.4805110 (2013).

4 Fried, D. V. *et al.* Prognostic value and reproducibility of pretreatment CT texture features in stage III non-small cell lung cancer. *Int J Radiat Oncol Biol Phys* **90**, 834-842, doi:10.1016/j.ijrobp.2014.07.020 (2014).

5 Hunter, L. A. *et al.* High quality machine-robust image features: identification in nonsmall cell lung cancer computed tomography images. *Med Phys* **40**, 121916, doi:10.1118/1.4829514 (2013).

6 Galloway, M. M. J. S. Texture analysis using grey level run lengths. **75**, 18555 (1974).

7 Tang, X. Texture information in run-length matrices. *IEEE Trans Image Process* **7**, 1602-1609, doi:10.1109/83.725367 (1998).

8 Ganeshan, B., Abaleke, S., Young, R. C., Chatwin, C. R. & Miles, K. A. Texture analysis of non-small cell lung cancer on unenhanced computed tomography: initial evidence for a relationship with tumour glucose metabolism and stage. *Cancer Imaging* **10**, 137-143, doi:10.1102/1470-7330.2010.0021 (2010).

9 Ganeshan, B. *et al.* Non-small cell lung cancer: histopathologic correlates for texture parameters at CT. *Radiology* **266**, 326-336, doi:10.1148/radiol.12112428 (2013).

10 Ganeshan, B., Panayiotou, E., Burnand, K., Dizdarevic, S. & Miles, K. Tumour heterogeneity in non-small cell lung carcinoma assessed by CT texture analysis: a potential marker of survival. *Eur Radiol* **22**, 796-802, doi:10.1007/s00330-011-2319-8 (2012).

11 Amadasun, M. & King, R. Textural Features Corresponding to Textural Properties. *Ieee T Syst Man Cyb* **19**, 1264-1274, doi:Doi 10.1109/21.44046 (1989).

12 Fave, X. *et al.* Impact of image preprocessing on the volume dependence and prognostic potential of radiomics features in non-small cell lung cancer. *Transl Cancer Res* **5**, 349-363, doi:10.21037/tcr.2016.07.11 (2016).

13 Zhang, L. *et al.* IBEX: an open infrastructure software platform to facilitate collaborative work in radiomics. *Med Phys* **42**, 1341-1353, doi:10.1118/1.4908210 (2015).

14 Shafiq-Ul-Hassan, M. *et al.* Intrinsic dependencies of CT radiomic features on voxel size and number of gray levels. *Med Phys* **44**, 1050-1062, doi:10.1002/mp.12123 (2017).
